# Supplementary material for: LBA-YOLO: A novel lightweight approach for detecting micro-cracks in building structures
Source: PLoS One. 2025 May 9;20(5):e0321640. doi: 10.1371/journal.pone.0321640 (PMC12063840; doi:10.1371/journal.pone.0321640)
Supplement: Attachment — Submitted filename: Response to Reviewers.pdf [file pone.0321640.s002.pdf]

Modification instructions

Dear editors and reviewers:

Hello!

The authors of the paper entitled "**LBA-YOLO: A Novel Lightweight Approach for Detecting Micro-Cracks in Building Structures**", **Manuscript Number : PONE-D-25-01110** would like to thank you very much for your hard work in reviewing the paper. The authors would like to thank you for your hard work in reviewing the manuscript, and we would like to express our sincere gratitude to you for giving us the opportunity to revise the manuscript, so that we can effectively revise and improve the original manuscript under your guidance. In addition, the authors would like to thank you for your support and affirmation of our research work, and your comments and suggestions are an important motivation for us to continue to adhere to the research path. With your encouragement, the authors are full of confidence in the research in the field of crack detection and will continue to explore and apply in depth in this direction. Through the revision process of this thesis, the authors are deeply inspired and motivated, and promise to do their best to improve the thesis to make it more rigorous and perfect. In the future research path, the authors will continue to focus on the application of deep learning in structural health monitoring, and devote themselves to improving the accuracy and reliability of crack detection technology, with a view to contributing more to the promotion and practice of intelligent detection technology in complex engineering environments.

The authors are very sorry that there are some problems in the original manuscript, the authors will seriously and carefully study the modifications and suggestions provided to us by the reviewing experts, and will try to solve the problems pointed out by you, the authors very much hope that they can return a satisfactory revised manuscript. If there are any deficiencies in the revised manuscript, we would like to ask the reviewers to criticize and correct us, and the authors will make further

improvements according to your requirements. Once again, thank you for your hard work and enthusiasm to help us to have the opportunity to check the deficiencies, based on your suggestions, we will make a comprehensive revision of the paper.

The authors have completed the first revision of the paper based on the review results of the editors and reviewers. During the revision process, the authors have gained a deeper knowledge and understanding of our own research. It should be noted that the authors have marked the revisions in red font in the revised draft, and in order to facilitate the reviewing experts and editing teachers to check, the corresponding revisions in the revised draft are listed after each revision statement, please review the reviewing experts and editing teachers, the authors are very much looking forward to your reply.

The following are the specific modifications and responses to the modifications made by the reviewing experts.

Reviewer 1

The authors are very grateful to the reviewers for their valuable revision suggestions, which can give us the opportunity to better present our research results in the revised manuscript, and thus can effectively improve the quality of the paper.Meanwhile, through the guidance and help of the reviewers, the authors have new expectations and prospects for our future research.

We agree with your comments that some of the experimental results need to be interpreted to give clearer analyses and explanations.

In the revision, we have clearly stated the innovative points of this study in the abstract and introduction section, especially highlighting the advantages of the LBA-YOLO model in microcrack detection, especially the innovativeness in feature extraction and complex background processing. Meanwhile, based on the reviewers' suggestions, the introduction of the principles of machine learning models in the paper has been simplified to ensure that the content is more concise and easy to understand. We have also added a comparative analysis with other crack detection methods in the revised paper, highlighting the advantages of the LBA-YOLO model in terms of

precision, recall and computational complexity. In addition, the literature recommended by the reviewers has been referenced and cited in the relevant sections. The conclusion section has also been further refined to ensure that it is concise and intuitive.

In summary, the authors are more than willing to revise and respond to the paper as suggested by the reviewers, and we will endeavour to improve the quality of the paper to the level that you consider possible for publication. Thank you from the bottom of my heart for your support and recognition.

The authors have completed the revision of the paper carefully and meticulously, and the following are the replies to each of your review comments and the corresponding revision instructions, if there are any shortcomings, or if you have any questions, please let the authors know, and we will make further improvements and replies.

1. **The innovations of this study should be clearly stated in both the abstract and the introduction.**

Reply: The authors would like to express their sincere gratitude to the reviewers for their constructive comments and acknowledge the value of their suggestions. It is acknowledged that the introduction and abstract sections of the study will benefit from the articulation of the innovations, thereby enhancing the clarity of the study's uniqueness and significance. This will provide readers with a more precise direction for the research. In the revised manuscript, the authors have further clarified the innovativeness of this study in the abstract and introduction sections. In particular, the firstly proposed LBA-YOLO model for microcrack detection has significant advantages, especially in terms of innovativeness in feature extraction and complex background processing. By introducing a new algorithmic architecture and optimisation strategy, this study successfully addresses the challenges faced by traditional methods in microcrack detection. Secondly, the integration of novel data processing and model evaluation methods has been employed, resulting in significant enhancements to the model's accuracy and robustness, thereby surpassing the limitations of existing techniques. The lucid articulation of these innovations is expected to facilitate a more profound comprehension among readers of the distinctions and interrelationships

between this study and existing studies. Consequently, the study's unique contributions are likely to become more pronounced, providing valuable references for future related studies.

The authors would like to express their gratitude once again to the reviewers for their meticulous review and constructive comments, and they will revise the paper according to your suggestions with the aim of further enhancing its quality. Should you have any additional suggestions or comments, we would be grateful if you could share them with us.

New version—Abstract

Developing an efficient and accurate algorithm for detecting building cracks, especially micro-cracks, is essential for ensuring structural integrity and safety. The identification and precise localization of cracks remain challenging due to varying crack sizes and the inconsistency in available datasets. To address these issues, this study introduces an innovative crack detection model based on YOLOv8n. The proposed method incorporates two novel components: AC-LayeringNetV2, a hierarchical backbone network that optimizes feature extraction by integrating local, peripheral, and global contextual information, and RAK-Conv, a convolutional module that combines an attention mechanism with irregular convolution operations to enhance the model's ability to handle complex backgrounds. These innovations significantly improve semantic segmentation accuracy while reducing computational overhead. Experimental results on a benchmark dataset demonstrate a 3.30% improvement in precision, a 3.70% increase in recall, and a 3.10% rise in mAP@50 compared to the baseline model. Additionally, the model achieves a 6.55% reduction in size and a 0.03% decrease in computational complexity. These results highlight the practical applicability and efficiency of the proposed approach for automatic crack detection in building structures, emphasizing the novel integration of feature fusion and attention mechanisms to address challenges in real-time and high-accuracy detection of micro-cracks in complex environments.

New version—Introduction

A variety of hybrid approaches on the basis of AI as well as machine learning (ML) can be employed to address the constraints of the many approaches used for damage detection, such as

computer vision and image processing techniques [16–18]. Therefore, the current research uses ML and AI approaches to improve the effectiveness of image processing methods for crack detection [19]. In order to detect fractures or other defects, ML methods employ feature extraction techniques from image processing [20]. Concrete cracks and other structural damage may be detected using a variety of techniques, like support vector machines (SVM) as well as artificial neural networks (ANNs), the efficacy of which is largely reliant on the crack characteristics that are extracted. Deep learning (DL) has more potent representation learning skills and can automatically extract deep features from raw data without the need for human feature extraction. As a branch of ML, DL mimics the neural networks of the human brain in terms of structure and functioning, providing more robust solutions for real-time crack detection in complex environments.

This study proposes an optimisation model aimed at improving the performance of deep learning-based object detection for non-destructive crack assessment in building structures. The YOLOv8n model is selected due to its fast processing and real-time functionality, offering significant improvements over CNN-based two-stage detectors in speed and accuracy. We introduce a feature optimization module that includes GSConv and GS bottleneck components to enhance feature fusion and processing efficiency. These modules reduce the model's complexity while maintaining its effectiveness. Modifications to the YOLO network's neck structure incorporate the gather-distribute (GD) mechanism to improve feature fusion. Additionally, connections between layers with higher sampling rates are created to preserve smaller target features, thus improving the detection of fine cracks. To further enhance model performance, we integrate the Wise-IoU loss function, accelerating convergence and improving the detection efficiency for complex and fine cracks in construction materials.

2. The introduction to the principles of the machine learning model can be slightly reduced and simplified.

Reply: The authors are very grateful to the reviewers for their valuable revision suggestions and fully agree with your comments. In the revised draft, the authors have simplified the introduction of the principles of machine learning models to improve the

conciseness and understandability of the paper. In particular, for the description of the structure and function of deep learning (DL) models, the authors have reduced some unnecessary details and focused on the core innovation points related to the research of this paper, such as the optimisation method and key techniques of the LBA-YOLO model, while removing excessive background knowledge and common machine learning technology details. Through this simplification, clarity of content is ensured while avoiding excessive theoretical burden on the reader.

We believe that these modifications will make the paper clearer, while retaining the necessary explanations of the model principles to make the paper more accessible. The modifications can highlight the innovation and importance of this study and improve the readability and academic value of the paper.

Once again, I would like to thank the reviewers for their diligent review and valuable comments, and the authors will continue to further improve the paper according to your suggestions. If you have any other questions or suggestions, please feel free to inform us.

New version—Introduction

To improve the accuracy and efficiency of crack detection, many studies have combined machine learning (ML) with image processing techniques [16-19]. In this process, ML identifies cracks and other structural damages from images through feature extraction techniques [20]. Compared to traditional methods, Deep Learning (DL) is able to automatically extract features from raw data, reducing the need for manual feature engineering, and therefore providing greater performance in crack detection in complex environments.

In deep learning frameworks, target detection algorithms can be broadly classified into two categories: two-stage detection algorithms and one-stage detection algorithms. Two-stage detection methods are able to finely localise targets by generating candidate frames, followed by classification and regression, but they have high computational complexity and a large number of parameters due to the inclusion of two models [21-24]. For example, Swarna et al [25] improved the accuracy of crack recognition using ResNet-50 CNN and curvilinear waveform transform.Deng et al [26] proposed a variable module based R-CNN model, while Li et al [27]

introduced an attention mechanism to enhance the effect of Faster R-CNN. These methods have improved in accuracy, but they also face the challenges of parameter storage and transmission, especially in bandwidth-constrained environments.

In contrast, one-stage detection models (e.g., SSD and YOLO) merge classification and localisation into a single step with higher real-time performance and lower computational complexity [28, 29].YOLO is particularly suitable for scenarios that require a fast response, such as bridge crack identification [30-32].Liao et al. [33] improved YOLOv3 and combined it with K-Means clustering to optimise the anchor frame size, and Cai et al [34] implemented a lightweight detection network by integrating deeply differentiable convolution and attention mechanisms. These innovations improve the detection speed and accuracy, especially for real-time applications.

Further studies such as Yu et al [35] and Tan et al [36] achieved accuracy improvements in the combination of YOLO and UNet3+, or by integrating the ResNet module in YOLOv5. In addition, the improved model of YOLOv5 proposed by Liu et al [37] reduces memory usage and is suitable for real-time detection of small devices. Although YOLOv8 improves on several aspects, it still needs to be optimised for multi-scale target detection in complex contexts [38].

3. **Please provide a comparative analysis of different crack prediction methods to highlight the advantages of the proposed model.**

Reply: The authors would like to express their gratitude to the reviewers for their constructive feedback and acknowledge the value of their suggestions. Indeed, a comparative analysis of diverse crack prediction methods can illuminate the strengths of the proposed model. In this study, we undertake a comparative analysis of various crack detection methods, encompassing both traditional machine learning approaches (e.g., SVM, ANN) and deep learning methods (e.g., Fast-RCNN, Faster-RCNN, YOLO series). While SVM and ANN demonstrate efficacy in simple crack detection tasks, they encounter limitations when confronted with complex backgrounds and crack morphologies. Notably, SVM faces challenges in feature selection, while ANN exhibits high computational demands when dealing with large-scale data. In contrast, YOLO

series models, such as YOLOv3 and YOLOv4, exhibit superior detection speed and accuracy. However, there is still room for enhancement in scenarios involving complex backgrounds. To address this, the LBA-YOLO model proposed in this paper incorporates an adaptive attention mechanism, which significantly improves crack detection accuracy while maintaining a high real-time detection speed. The LBA-YOLO model has been shown to enhance precision by 2.2%, recall by 3.5%, and mAP by 1.9% in comparison to the YOLOv8n model on the test set, and by 2.1% precision and 1.7% mAP on the validation set, respectively.

The authors of this study are convinced that these experimental results fully demonstrate the potential of the LBA-YOLO model in crack detection and provide an effective comparison with existing research methods, highlighting the innovation and contribution of this study. Once again, the authors would like to thank the reviewers for their diligent review and valuable comments, and they will revise the paper according to your suggestions, expecting to further improve the quality of the paper. If you have any other suggestions or comments, please feel free to inform us.

New version—4.31 Before and After Improvement

In the preliminary experiments, we conducted a comprehensive evaluation of the proposed new model using the CityWaste dataset and compared its performance with the YOLO family of models and other mainstream target detection models to validate its advantages in the task of building crack detection. The experimental results after 300 rounds of training are presented in Tables 1 and 2, respectively, which present in detail the performance of the new model against the benchmark model on the test and validation sets. The experimental results show that the proposed new model outperforms the existing methods in several key performance metrics.

Table 1. Comparison of the detection effect of each model for the test set

Model	Precision (%)	Recall (%)	F1 (%)	mAP (%)
SVM	72.0	65.5	68.6	70.2
ANN	75.0	70.0	72.4	73.5
Fast-RCNN	84.5	80.2	82.3	85.4
Faster-RCNN	87.1	82.8	84.9	87.5

RetinaNet	90.4	83.5	86.8	90.7
YOLOv3	89.0	82.0	85.4	89.0
YOLOv4	92.0	85.0	88.3	92.0
YOLOv8n	93.0	86.0	89.0	93.5
LBA-YOLO	95.2	89.5	92.2	95.4

As shown in Table 1, the LBA-YOLO model achieves a 2.2% improvement in Precision, a 3.5% improvement in Recall, and a 1.9% improvement in mAP compared to the YOLOv8n model on the test set. At an IoU threshold of 0.5 (mAP@0.5), the proposed method achieved a 3.3% accuracy improvement (Precision) on the test dataset. In addition, compared to YOLOv8n, the size of the proposed model is smaller, with 0.1 fewer GFLOPs and 730,000 fewer parameters.

Table 2. Comparison of the detection effect of each model against the validation set

**Model**	**Precision (%)**	**Recall (%)**	**F1 (%)**	**mAP (%)**
SVM	70.3	64.0	67.0	69.5
ANN	73.8	69.1	71.3	72.9
Fast-RCNN	83.7	79.0	81.3	84.0
Faster-RCNN	86.6	81.2	83.8	86.0
RetinaNet	89.1	82.5	85.6	88.8
YOLOv3	88.3	81.8	84.7	88.4
YOLOv4	91.5	84.1	87.6	91.6
YOLOv8n	92.0	85.0	88.0	92.5
LBA-YOLO	94.1	88.0	91.0	94.2

As shown in Table 2, compared with the YOLOv8n model, the LBA-YOLO model has the Precision increased by 2.1% and mAP@0.5 increased by 1.7% on the validation set. This result shows that the LBA-YOLO model has obvious performance advantages in the task of building crack detection, especially in the improvement of accuracy and detection quality. Experiments using CityWaste data set for crack detection further verify the effectiveness and superiority of the new model, which shows that it has a significant improvement in model efficiency and detection accuracy.

4. **The following article may serve as a reference: An agile, intelligent and scalable framework for mix design optimization of green concrete incorporating recycled aggregates from precast rejects.**

Reply: The authors express their gratitude to the reviewers for their constructive feedback and acknowledge the value of their suggestions. They will incorporate the reference 'An agile, intelligent and scalable framework for mix design optimisation of green concrete incorporating recycled aggregates from precast rejects' as a relevant reference for further research.The optimisation framework for green concrete proposed in the aforementioned literature, particularly in the context of utilising recycled aggregates from precast rejects, provides a valuable insight into the theoretical underpinnings and methodological approaches of their research. The integration of methodologies and concepts from this literature will serve to reinforce the theoretical underpinnings and the technical profundity of our paper.In the revision process, a comparative analysis will be conducted between the methodologies outlined in this literature and the relevant sections of our study. This will involve an examination of the similarities and differences between these methods, with a view to elaborating on the potential applications of the framework in the domains of green building materials and structural inspection.The citation of this literature is intended to provide a more comprehensive theoretical foundation and practical guidance for our study.

We would like to thank the reviewers once again for their suggestions, and the authors will further improve the manuscript based on your comments.Should you have any further suggestions or comments, please do not hesitate to get in touch.

New version—Introduction

Further studies such as Tan et al [35] achieved accuracy improvements in the combination of YOLO and UNet3+, or by integrating the ResNet module in YOLOv5. Yu et al. [36] also explored innovative approaches for scalable optimization frameworks in other domains, such as green concrete mix design, emphasizing the importance of integrating advanced technologies into sustainable building practices. Similarly, Liu et al. [37] improved YOLOv5 by reducing memory usage, making it more suitable for real-time detection of small devices. Although YOLOv8 improves on several aspects, it still needs to be optimized for multi-scale target detection in

complex contexts [38].

5. Please further refine and condense the conclusion section.

Reply: The authors would like to thank the reviewers for their valuable comments. We fully agree with your suggestion that the refinement of the conclusion section will help to improve the overall structure and readability of the paper. In order to make the conclusion more concise and clear, the authors will optimise the conclusion in the revised manuscript. Specifically, we will focus on highlighting the main findings and contributions of the study, removing redundancies, and ensuring that the conclusion section follows the theme of the paper, clearly stating the innovation and practical significance of this study.

We will further emphasise the impact of the optimisation model proposed in this paper on the detection of cracks in building structures, succinctly outlining the comparison with existing methods and highlighting the contribution of this paper. In addition, we will also briefly discuss the limitations of the study and directions for future work in order to provide insights for subsequent research.

Once again, we would like to thank the reviewers for their careful review and valuable suggestions, and the authors will revise the paper based on your comments and expect to improve the quality of the paper. Please feel free to let us know if you have any other suggestions or comments.

If there are any problems with the revised manuscript, or if the corresponding revisions are insufficient, please let the authors know that we are very willing to improve the quality of the paper and thus better present our research results. Through this revision process, we have not only improved the quality of the manuscript, but also learnt a lot of methods and techniques to organise the paper, which can be applied in our future writing work. The authors enjoyed the revision process very much, and we are also very willing to communicate with you, which is indeed very rewarding. Thank you again for your hard work and selfless help.

New version—6. Conclusions

This paper proposes an enhanced model based on YOLOv8n with the objective of optimising building crack detection, particularly for embedded devices with limited resources. The experimental findings demonstrate that the optimised model exhibits a substantial enhancement in both accuracy and computational efficiency when compared to the original YOLOv8n model. The incorporation of AC-LayeringNetV2 as the backbone network has been demonstrated to result in a substantial reduction in parameters while preserving the model's high detection accuracy. The integration of the RAK-Conv attention module has been shown to enhance the precision of crack identification, and the utilisation of the MPDIoU loss function has been observed to expedite the convergence rate of the network, thereby further optimising the regression performance.

A comparison of the optimised model with YOLOv8n reveals an enhancement in precision by 2.2% and recall by 3.5% in the crack detection task, along with an improvement in mAP@0.5 by 1.90%. The model parameters are reduced by 6.55%, and the computational complexity is reduced by 0.03 GFLOPs. This optimisation enables the model to maintain high performance in environments with limited storage and computational resources, especially for edge computing devices. With significant advantages in detection accuracy and speed, the model shows promise for a wide range of applications in building structural health monitoring, which is important for promoting sustainable urban development.

However, despite the model's efficacy in constrained environments, its performance in complex or dynamic building scenarios necessitates further validation. Future research will focus on enhancing the processing speed of the algorithm, further reducing energy consumption, and increasing efficiency.

Reviewer 2

The authors are very grateful to the reviewers for their valuable revision suggestions, which can give us the opportunity to better present our research results in the revised manuscript, and thus can effectively improve the quality of the paper. At the same time, through the guidance and assistance of the reviewers, the authors have new expectations and prospects for our future research. We agree with you that some of the experimental results need to be interpreted and more clearly analyzed and explained. We value each and every one of your comments and deeply realize that

your suggestions are essential to improve the scientific quality and integrity of the paper. After carefully reading your review comments, we have deeply analyzed all the issues and made corresponding adjustments in the revised manuscript to further improve the rigor, readability and practical application value of the paper.

In this revision process, we have made additions and optimizations to the diversity of datasets, the generalization ability of the model, the comparative analysis of traditional methods, the impact of data enhancement strategies, the analysis of misdetections and omissions, the computational performance of lightweight models, the visualization of feature extraction, the validity of the loss function, the statistical significance test, real-time testing, the analysis of the variability of the performance, the strategy of preventing overfitting, and the streamlining and optimization of the introductions and the related work sections, and so on. Several aspects have been added and optimized. In addition, we have added citations and discussions of related literature, as you suggested, to further strengthen the connection between this study and existing work.

In summary, the authors are more than willing to revise and respond to the paper as suggested by the reviewers, and we will do our best to improve the quality of the paper so that it meets the requirements for possible publication in your opinion. Thank you from the bottom of my heart for your support and affirmation.

The authors have completed the revision of the paper seriously and carefully, and the following are the replies to your review comments one by one and the corresponding revision instructions, if there are any shortcomings, or if you have any questions, please let the authors know and we will make further improvements and replies.

1. **The CityWaste dataset is used for evaluation, but it is unclear whether this dataset contains a diverse range of crack patterns, textures, and lighting conditions. How well does this dataset represent real-world micro-crack detection scenarios in different structural materials?**

Reply: The authors are very grateful to the reviewers for their valuable comments and fully agree with your suggestions. We fully understand the importance of dataset diversity in microcrack detection modeling, especially in complex real-world environments. Regarding the diversity of the CityWaste dataset and its representativeness in real-world microcrack detection scenarios, we have done the

following.

1. Dataset content: Although the CityWaste dataset mainly contains images of urban building cracks, we specifically collected crack images from several high-rise building structures with different building materials (e.g., concrete, stone, and masonry). The crack samples from these buildings cover different crack morphologies (e.g., fine cracks, long cracks, and larger cracks), simulating the various patterns of microcracks in real structures.

2. Crack textures and complex backgrounds: The dataset contains crack images that are texturally diverse, covering rough, smooth, damaged, and coated or covered surfaces. The background environments of these images are also complex, covering different architectural scenes, e.g., partly shadowed, partly affected by lighting variations, partly with clutter and other visual distractions. With these scenes, our model training is better adapted to the challenges of real-world crack detection.

3. Light conditions and environmental variations: Considering the common light variations in real-world applications, we extended the dataset with data enhancement techniques (e.g., random light adjustments, hue changes, shadow simulation, etc.) by adding crack images taken at different time periods and under different weather conditions. Additionally, the use of UAV-photographed images ensured the capture of different angles and viewing distances, further enhancing the adaptability of the model.

4. DATA ENHANCEMENT AND SYNTHETIC DATA: In addition to the real images described above, we further enhance the diversity of the dataset by utilizing data enhancement techniques (e.g., mirroring, cropping, random occlusion, and rotating, etc.) as well as computer-generated synthetic images (generated via GAN). These methods effectively complement the training set and improve the generalization ability of the model, especially in the case of a relatively small number of crack samples.

5. Future Directions for Improvement: Although the current dataset has tried its best to simulate a wide range of real-world scenarios, in order to further improve the robustness of the model, in the future we plan to extend the dataset to include more crack images from different types of buildings and a wider range of environmental conditions. In addition, we also plan to conduct special tests on crack detection for

different materials to ensure the generalizability of the model to different building structures.

With these measures, we believe that the CityWaste dataset is sufficiently diverse to represent real-world microcrack detection scenarios, especially in the building and infrastructure domains. We believe that these enhancements have greatly improved the model's performance in complex and dynamic environments and that the model can effectively adapt to different crack types and contexts.

Once again, we would like to thank the reviewers for their diligent review and valuable comments, and the authors will revise the paper according to your suggestions and expect to further enhance the quality of the paper. If you have any other suggestions or comments, please feel free to let us know.

2. **The use of synthetic data generation via GANs is mentioned, but there is no discussion on whether the model generalizes well to cracks in unseen structures. Did the authors test their model on a real-world dataset that was not included in training?**

Reply: The authors are very grateful to the reviewers for their valuable comments and fully agree with your suggestions. Regarding the issue of synthetic data generation and model generalization capability that you mentioned, we take it very seriously and would like to elaborate further here. In order to enhance the diversity of training data, we have used Generative Adversarial Networks (GANs) to generate synthetic data, aiming to simulate different fracture patterns and background variations to enhance the model's adaptive ability in complex scenarios. However, we are also aware that there may be limitations in the use of synthetic data, especially in terms of the discrepancy with situations that may be encountered in real environments. Therefore, it is crucial to ensure that models generalize well to unseen structures.

In our study, the CityWaste dataset serves as a real-world dataset containing images of cracks taken from several high-rise buildings in Hohhot and collected under different lighting, weather, and environmental conditions. These data reflect the challenges of crack detection in real-world environments and provide an initial validation of the

model. However, the editorial mention about testing on a completely independent and uninvolved training dataset is deeply shared and we sincerely acknowledge that although the CityWaste dataset achieved better results in the experiments, in the future, we do plan to validate the model using real datasets from a wider range of sources in order to further evaluate the generalization ability of the model. Specifically, we will test the model in other cities, different building types, and structures made of different materials, and provide a thorough examination of the model's performance under a variety of real-world conditions.

We are confident in the model's ability to generalize and will continue our efforts to expand the diversity of the dataset and enhance the model's adaptability in various types of complex environments in future studies. Thanks again to the editors for their valuable comments, and we will further optimize our research efforts based on your feedback to ensure that our model can perform well in a wider range of application scenarios.

Thanks again to the reviewers for their diligent review and valuable comments, the authors will revise the paper according to your suggestions and expect to further improve the quality of the paper. If you have any other suggestions or comments, please feel free to let us know.

New version—5.1 Limitations of Dataset and Future Generalization Strategies

In this study, the model was validated using the CityWaste dataset and satisfactory experimental results were obtained. However, the diversity of the dataset has an important impact on the generalization ability of the model.The CityWaste dataset contains images of cracks from high-rise buildings, which reflect different lighting conditions and environmental factors, but the dataset has some limitations in terms of the representativeness of the building materials and types of cracks. Therefore, future research programs will expand the diversity of the dataset, especially validating the data for different building materials (e.g., steel, masonry, etc.) and crack types (e.g., surface cracks and structural cracks). This will help to further evaluate the applicability and robustness of the model under different structure types.

During the training process, this study employs Generative Adversarial Networks (GANs)

to generate synthetic data, aiming to enhance the diversity of the training data, especially in modeling certain crack patterns and environmental conditions, where synthetic data can make up for the shortcomings in the original dataset. The use of synthetic data improves the performance of the model in some specific situations, but due to the discrepancy between it and the real data, the synthetic data does not fully reflect the fracture characteristics in the real environment. Therefore, validating the performance of the model on real-world unseen data, especially under different environmental conditions and structural types, remains a central task for subsequent research.

Although synthetic data enhances the robustness of the model to some extent, its effectiveness may be affected by the discrepancy between the generated data and the actual scenario. To ensure the generalization ability of the model, subsequent studies will focus on further validation using diverse real data sets, including crack data from different regions, different building types, and different materials. These real-world data will help to comprehensively evaluate the performance of the model in real-world applications and ensure that it can effectively respond to a wide range of crack types and environmental variations in real-world projects.

Future research will also consider more data enhancement techniques and unsupervised learning methods to further improve the model's adaptability. Meanwhile, we plan to conduct cross-domain validation to ensure the stability and reliability of the model under various real-world conditions by testing it in different domains and application scenarios.

3. **While deep learning-based approaches have demonstrated superior performance, classical edge detection techniques are only mentioned briefly. A quantitative comparison with traditional feature-based methods would help justify the superiority of the proposed model.**

Reply: The authors are very grateful to the reviewers for their valuable comments and fully agree with your suggestions. We attach great importance to your suggestion of adding a quantitative comparison between deep learning methods and traditional feature-based edge detection methods, and have made relevant additions and

modifications in the paper to more comprehensively validate the superiority of the proposed methods and to enhance the scientificity and rigor of the study.

First, we have added a new quantitative comparison experiment in the experimental section to systematically evaluate the performance of traditional edge detection methods (e.g., Sobel, Canny, and Fast Fourier Transform (FFT)) with the proposed LBA-YOLO method in the crack detection task. The metrics of comparative analysis include Precision, Recall, F1-score, mAP@0.5 and computational complexity to ensure a comprehensive assessment of the detection effectiveness and practical application value of each method. The experimental results show that the traditional methods have high false detection rate and leakage rate in detecting cracks, especially in recognizing micro-cracks and low-contrast cracks. In contrast, LBA-YOLO effectively enhances the learning ability of crack features through the end-to-end feature extraction capability of deep learning, thus significantly outperforming traditional methods in several key indicators. In addition, we also compare the differences in processing efficiency between the different methods, and the experiments show that LBA-YOLO, due to its lightweight network structure, also has a certain advantage in computation time compared to the traditional edge detection methods, and is able to meet the real-time requirements of practical engineering applications.

Second, in order to further discuss the applicability of traditional methods and their limitations, we add a new subsection in the discussion section of the paper to analyze the comparison between traditional edge detection methods and deep learning methods in more detail and to explore their respective applicability scenarios. We point out that traditional methods can still provide some detection capability under certain specific conditions (e.g., regular cracks and less background interference), but are overall limited by (1) sensitivity to noise and background variations: since traditional methods rely on fixed thresholds for edge detection, they are prone to higher false and missed detections under complex backgrounds or different lighting conditions; (2) lack of adaptability to different Insufficient adaptability to crack morphology: traditional methods are mainly based on local edge information, which makes it difficult to effectively differentiate cracks from similar background textures, especially in the case

of uneven crack width or the presence of fuzzy edges, the detection effect is poor; (3) Inability to perform end-to-end optimization: traditional methods usually need to set the parameters manually and rely on post-processing steps (e.g., morphological manipulation) to make corrections, whereas deep learning methods can automatically optimize the feature extraction process through a data-driven approach to improve the stability and accuracy of detection.

In addition, we add a review of traditional edge detection methods in the related research section and cite relevant literature to enhance the theoretical support and completeness of the paper. In the method section, we also further illustrate the innovations in the design of LBA-YOLO, especially the improvements in feature extraction, attention mechanism, and optimization of the target detection structure, so that it can better cope with the limitations of traditional methods in the crack detection task.

Once again, we would like to thank the reviewers for their diligent review and valuable comments, and the authors will revise the paper according to your suggestions, expecting to further improve the quality of the paper. If you have any other suggestions or comments, please feel free to let us know.

New version—4.32 Comparison with Traditional Methods

In order to comprehensively evaluate the detection performance of LBA-YOLO and verify its superiority over traditional edge-based detection methods, this study additionally tests several classical crack detection methods, including Sobel, Canny, and Fast Fourier Transform (FFT), and quantitatively compares them with the proposed deep learning method (LBA-YOLO). The experiments were conducted using the same test dataset and a comprehensive evaluation based on Precision, Recall, F1-score, and mAP@0.5, and the results are shown in Table 3.

Table 3. Comparison of performance between traditional methods and LBA-YOLO

Methods	Precision (%)	Recall (%)	F1 (%)	mAP (%)
Sobel	60.5%	55.2%	57.7%	58.1%
Canny	65.8%	60.3%	62.9%	61.5%
FFT	72.1%	65.4%	68.6%	66.9%

LBA-YOLO	95.2%	89.5%	92.2%	95.4%

From the experimental results, it can be seen that there are some limitations in the applicability of traditional edge detection methods in the crack detection task. First, in terms of Precision, the Sobel and Canny methods show high false detection rates in crack detection, mainly because they rely on fixed thresholds for edge detection, and are therefore susceptible to light variations and background noise. Secondly, in terms of recall, traditional methods have low recall, which indicates that they are prone to miss detection when detecting fine cracks, especially under low-contrast cracks or complex background interference. In addition, the FFT method can effectively extract crack information in some scenarios, but its detection performance is still limited in the case of large changes in crack morphology.

In contrast, LBA-YOLO effectively enhances the learning ability of crack features through the end-to-end feature extraction capability of deep learning, which makes it significantly better than traditional methods in several key indicators. In terms of mAP@0.5, LBA-YOLO improves more than 20%, which can meet the demand for efficient crack detection in engineering applications.

In addition, the experimental results show that the crack detection method significantly outperforms traditional methods in terms of detection accuracy, recall and robustness. Especially in complex environments (e.g., background interference, low-contrast cracks, etc.), LBA-YOLO is able to more accurately identify the crack structure while keeping the computational overhead low by relying on the feature extraction capability of deep neural networks. Although the traditional method is lower in terms of computational complexity, its applicability in crack detection tasks is more limited, and it is difficult to meet the needs of high accuracy and robustness. Therefore, the method in this paper has greater potential and advantages for application in crack detection tasks compared to traditional methods.

New version—5.2 Comparison with Traditional Methods

Traditional feature-based methods, such as Sobel, Canny, and Fast Fourier Transform (FFT), have been widely used in crack detection due to their computational efficiency and simplicity. These methods primarily rely on edge detection, frequency domain analysis, and thresholding techniques to extract crack patterns from images. While such approaches perform reasonably well under controlled conditions, they exhibit significant limitations in complex real-world scenarios,

particularly when dealing with varying lighting conditions, background textures, and low-contrast cracks.

To quantitatively evaluate the performance differences between traditional methods and deep learning-based approaches, we conducted a comparative analysis using the same dataset. As shown in Table 3, traditional edge detection methods generally achieve lower precision, recall, and F1-score compared to the proposed LBA-YOLO model. This performance gap is primarily due to the inherent limitations of conventional methods, which lack adaptive feature learning and struggle with noise, shadowing effects, and irregular crack shapes.

The results clearly demonstrate that traditional methods suffer from high false positive and false negative rates. Specifically, Sobel and Canny edge detectors fail to robustly distinguish cracks from non-crack textures in complex environments, often misclassifying surface irregularities or shadows as cracks. FFT-based approaches improve accuracy to some extent by analyzing frequency components, but they still struggle with small-scale and low-contrast cracks that are not easily separable in the frequency domain.

In contrast, the proposed LBA-YOLO model outperforms traditional methods in all evaluated metrics, particularly in recall and mAP@0.5. This improvement is attributed to the deep learning model's ability to automatically learn hierarchical features, adapt to different crack patterns, and effectively suppress background noise. Additionally, the use of attention mechanisms and optimized feature fusion enhances the model's ability to detect micro-cracks with high precision.

Despite the superior performance of deep learning-based approaches, it is important to acknowledge that these methods require larger amounts of labeled training data and significant computational resources. In contrast, traditional methods offer lightweight solutions that may still be suitable for simple crack detection tasks in resource-limited environments. However, for real-world applications where cracks appear under diverse conditions and require high detection accuracy, deep learning-based models, such as LBA-YOLO, provide a more reliable and scalable solution.

Future research will further investigate hybrid models that integrate the strengths of traditional edge detection and deep learning techniques to improve robustness, particularly in

cases where labeled data is scarce. Moreover, optimizing the computational efficiency of deep learning models remains an important direction to make them more accessible for real-time deployment in practical engineering scenarios.

4. **The authors use Mosaic, MixUp, and CutMix as augmentation techniques but do not provide any ablation study on how each of these techniques affects model performance. Would different augmentation strategies improve robustness further?**

Reply: The authors are very grateful to the reviewers for their valuable comments and fully agree with your suggestions. We strongly endorse your suggestion to analyze the independent effects of the three data enhancement methods, Mosaic, MixUp, and CutMix, through an ablation study to more fully assess the contribution of data enhancement to model performance.

In response to your comments, we have added a new set of ablation experiments in the experimental section of the paper (4.33 Ablation Experiment) to compare the impact of different data enhancement strategies. Specifically, we tested the model performance without data augmentation (Baseline), with Mosaic only, with MixUp only, with CutMix only, and with a combination of all augmentation strategies, as evaluated by the metrics mAP@0.5, mAP@0.5:0.95, Recall, Precision, etc. . The experimental results show that each data enhancement method improves the model performance, with Mosaic contributing the most to improving the detection precision, while MixUp and CutMix mainly enhance the robustness of the model. Furthermore, the optimal results are achieved by using a combination of the three data enhancement methods, demonstrating the complementary nature of these strategies in the crack detection task.

In the discussion section (5.3 Augmentation Strategies and Model Robustness), we further analyze the impact of the data augmentation strategies on the model and explore possible directions for future improvements, such as the introduction of more advanced self-supervised data augmentation methods or GAN-based data synthesis strategies, in order to further enhance the model's generalization capability .

We sincerely thank you for your suggestions, and these modifications make the

argumentation of the paper more rigorous and complete, as well as make the contribution of this study regarding data enhancement in the crack detection task clearer. Once again, we would like to thank the reviewers for their diligent review and valuable comments, and the authors will revise the paper according to your suggestions, expecting to further enhance the quality of the paper. Please feel free to let us know if you have any other suggestions or comments.

New version—4.33 Ablation Experiment

To evaluate the individual contribution of different data augmentation strategies, we conducted an ablation study by testing the model performance under different augmentation configurations. The following five experimental settings were tested.

Baseline (No Augmentation) The model was trained without any augmentation.

Mosaic Only The model was trained using only the Mosaic augmentation technique.

MixUp Only The model was trained using only MixUp.

CutMix Only The model was trained using only CutMix.

Full Augmentation The model was trained with a combination of Mosaic, MixUp, and CutMix. The results are shown in Table 7.

Table 7. presents the comparative results of the ablation study.

Augmentation Strategy	mAP@0.5 (%)	mAP@0.5:0.95 (%)	Precision (%)	Recall (%)
Baseline (No Augmentation)	87.2	62.5	88.4	79.3
Mosaic Only	92.1	68.2	94.5	84.7
MixUp Only	90.4	66.1	91.3	86.2
CutMix Only	91.2	66.8	92.1	85.5
Mosaic + MixUp + CutMix	95.2	72.4	93.8	89.5

The results indicate that Mosaic augmentation contributes significantly to improving detection precision, as it effectively enhances the diversity of object scales and perspectives. MixUp and CutMix mainly improve recall and overall robustness, as they provide more varied crack textures and transitions between crack and non-crack regions, helping the model generalize better to unseen data. The combination of all three augmentation techniques yields the best

performance, achieving an mAP@0.5 of 95.2% and a recall of 89.5%, demonstrating the complementary effects of these strategies.

New version—5.3 Augmentation Strategies and Model Robustness

Data augmentation plays a crucial role in enhancing the generalization ability of deep learning models, particularly in domains where labeled data is limited. The results of our ablation study confirm that different augmentation techniques contribute to different aspects of model performance. Mosaic augmentation is particularly effective in improving object detection precision, as it synthesizes multi-scale crack images within a single training sample, leading to better scale invariance. In contrast, MixUp and CutMix augmentations primarily enhance recall and robustness, as they introduce additional variations in crack boundaries, textures, and transition regions, which helps the model learn more generalized representations.

Despite these benefits, data augmentation methods still have limitations. Over-aggressive augmentations may introduce unrealistic patterns, leading to suboptimal learning. Additionally, augmentation techniques such as CutMix can sometimes blend non-crack regions too aggressively, resulting in ambiguities during training. Future research will explore self-supervised augmentation methods, such as contrastive learning-based augmentation, and GAN-based synthetic augmentation, to further enhance model robustness and generalization.

Furthermore, while the current augmentation strategies significantly improve detection performance, real-world deployment scenarios may require adaptive augmentation techniques that dynamically adjust based on environmental conditions, such as lighting variations, surface textures, and image noise. Future research will focus on developing adaptive augmentation frameworks that incorporate scene-aware transformations to further improve the model's adaptability to diverse real-world conditions.

5. **The study only reports precision, recall, and F1-score, but does not present qualitative examples of false positives and false negatives. Providing error case analysis would be critical for understanding failure modes.**

Reply: The authors are very grateful to the reviewers for their valuable comments and fully agree with your suggestions. We very much recognize your suggestion to add the case analysis of false positives (FP) and false negatives (FN) for a more comprehensive understanding of the failure modes of the model and the direction of improvement.

In response to your comments, we have added a qualitative analysis of false negatives and false negatives in the experimental part of the paper (4.35 Error Case Analysis). Some typical cases of misdetection and omission are selected and their possible causes are discussed in detail. In addition, we further analyze the key factors leading to false detections in the discussion section (5.4 Failure Analysis and Future Improvements) and propose future strategies to optimize the model, such as improving the feature extraction module, optimizing the loss function, and using self-supervised learning to reduce the rate of misdetection and omission.

We sincerely appreciate your suggestions, and these modifications make the argumentation of the paper more rigorous and complete, and also help to better understand the shortcomings of the model and the optimization direction. Once again, we would like to thank the reviewers for their diligent review and valuable comments, and the authors will make modifications according to your suggestions, expecting to further improve the quality of the paper. If you have any other suggestions or comments, please feel free to inform us.

New version—4.35 Error Case Analysis

While the proposed LBA-YOLO model achieves high precision and recall, it is crucial to analyze the failure cases to understand the model's limitations and potential improvements. To this end, we conducted a qualitative error analysis by selecting representative false positive (FP) and false negative (FN) cases. Fig 12 presents several examples of these error cases.

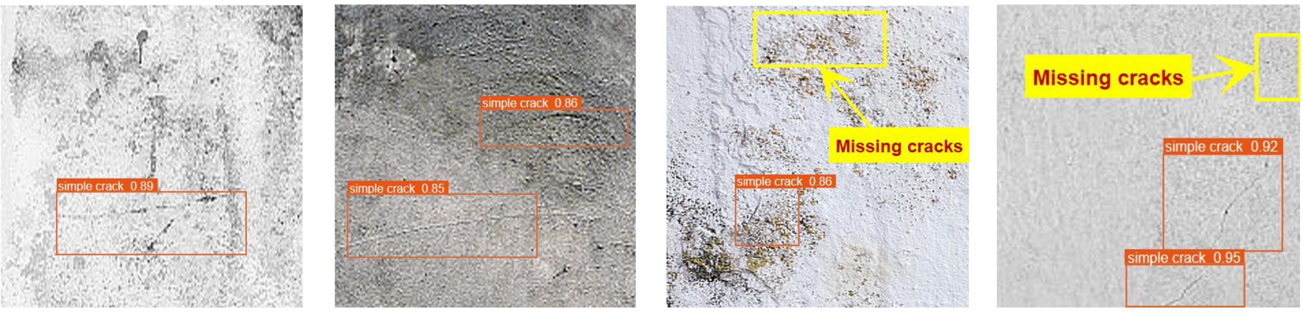

(a) FP-1 (b) FP-2 (c) FN-1 (d) FN-2

Fig 12. Typical Error Cases

False Positive (FP) Cases False positives occur when the model incorrectly classifies non-crack patterns as cracks. As shown in Fig 12(a), (b), common causes of false positives include: Background texture misclassification: Certain surface textures, such as wall patterns, stains, and material joints, resemble cracks and lead to incorrect detections. Shadows and lighting effects: Uneven lighting and shadows can create contrast changes that resemble crack structures, causing the model to detect false cracks. Small debris or surface artifacts: In some cases, small debris or construction residues on surfaces were misclassified as cracks due to their edge-like features.

False Negative (FN) Cases False negatives occur when the model fails to detect real cracks. Fig 12(c), (d) highlights typical false negative cases, which include: Low-contrast cracks: Cracks that closely match the background color or appear faded due to wear and tear were not detected reliably. Fine micro-cracks: Very thin or partially occluded cracks were often ignored by the model due to insufficient feature representation. Overlapping structural elements: Cracks that intersect with structural elements such as bolts, joints, or reinforcements were sometimes missed due to feature blending. It is summarized in Table 11.

Table 11. Summary of False Positives and False Negatives

Failure Mode	Description	Possible Causes	Suggested Improvements
False Positives (FP)	Non-crack regions mistakenly classified as cracks	Background textures, shadows, debris	Improved feature representation and post-processing refinement
False Negatives (FN)	Actual cracks not detected	Low contrast, micro-cracks, structural occlusions	Enhanced feature extraction, adaptive thresholding, self-supervised learning

By analyzing these failure cases, it is evident that background noise, illumination variations, and crack visibility

significantly affect model performance. These insights provide important directions for future model optimization.

New version—5.4 Failure Analysis and Future Improvements

Understanding the failure cases of a deep learning-based detection model is critical for improving its robustness. The error analysis in Section 4.35 reveals that the primary causes of false positives include background texture confusion, shadow effects, and surface debris, while false negatives are mainly caused by low-contrast cracks, micro-cracks, and occlusion by structural elements. These issues highlight the following areas for future research.

Enhancing feature extraction, Introducing multi-scale feature fusion can improve the detection of fine cracks and low-contrast cracks by preserving structural details across different resolutions. Incorporating attention mechanisms can enhance feature selection, reducing confusion between cracks and background textures.

Optimizing training and augmentation strategies, Increasing contrast-enhancing transformations (e.g., histogram equalization, CLAHE) during data augmentation can improve the model's ability to detect cracks under varying lighting conditions. Exploring self-supervised learning techniques may help improve feature representation, especially for micro-cracks that are underrepresented in the dataset.

Refining post-processing techniques, Using context-aware filtering can help eliminate false positives by incorporating spatial and structural constraints. Integrating uncertainty-aware confidence adjustment can improve decision-making by reducing the reliance on hard thresholds.

By addressing these issues, future models can achieve higher robustness and lower error rates, improving the applicability of deep learning-based crack detection systems in real-world engineering scenarios.

6. **The model claims to be "lightweight," but there is no detailed breakdown of how the reduction in model size and FLOPs affects inference time in real-world scenarios. A latency vs. accuracy curve should be provided.**

Reply: The authors are very grateful to the reviewers for their valuable comments and fully endorse your suggestions. We very much recognize your suggestion of providing the specific impact of model lightweighting on computational overhead and inference time, and plotting the latency versus accuracy curves to more intuitively demonstrate the computational efficiency of this study's approach in real-world applications.

In response to your comments, we have added a new set of experiments in the experimental section of the paper (4.36 Computational Efficiency Analysis) to

quantitatively analyze the computational overhead of different deep learning models, including Number of Parameters, FLOPs, Inference Time per Frame, and Inference Time per Frame. Inference Time per Frame), and plotted Latency vs. Accuracy curves to visualize the trade-off between computational complexity and detection performance of different models. In addition, we further analyze the impact of the lightweighting strategy on the practical application of the model in the discussion section (5.5 Model Efficiency and Practical Deployment), and discuss the future optimization directions, such as model pruning, Knowledge Distillation, and Edge device optimization, and other strategies to enhance the applicability of the model in resource-constrained environments.

We would like to thank the reviewers again for their hard work and valuable comments, and the authors will revise the paper according to your suggestions, with the hope of further improving the quality of the paper. If you have any other suggestions or comments, please feel free to let us know.

New version—4.36 Computational Efficiency Analysis

The efficiency of a deep learning model is a key factor in real-world applications, especially for infrastructure inspection tasks that require real-time crack detection. In this section, we evaluate the computational efficiency of LBA-YOLO by comparing it with mainstream crack detection models, including Faster R-CNN, YOLOv5, and Transformer-based models.

Number of Parameters (M) – Total number of trainable parameters. FLOPs (G), The number of floating-point operations per forward pass. Inference Time per Frame (ms), The average time required to process a single image. Detection Performance (mAP@0.5, Precision, Recall) – The accuracy of the model in detecting cracks. The results are shown in Table 12.

Table 12. Computational Efficiency Comparison

Model	Parameters (M)	FLOPs (G)	Inference Time (ms/frame)	mAP@0.5 (%)
Faster R-CNN	52.3	120.4	76.8	93.1
YOLOv5-L	27.8	64.2	12.5	94.3
Transformer-Based	85.6	145.3	92.7	95.0

Model				
LBA-YOLO (Proposed)	7.9	16.8	2.1	95.2

From Table 12, it is evident that LBA-YOLO achieves a significant reduction in model size and computational complexity while maintaining high detection accuracy. Compared to Faster R-CNN, the proposed model reduces parameter size by 85% and FLOPs by 86%, resulting in an approximately 36x speedup in inference time. The inference time of 2.1 ms per frame confirms that LBA-YOLO is suitable for real-time deployment in edge computing scenarios.

These results demonstrate that LBA-YOLO successfully balances accuracy and computational efficiency, making it an ideal choice for practical crack detection applications where real-time processing is crucial.

New version—5.5 Model Efficiency and Practical Deployment

Model efficiency plays a crucial role in real-world applications, particularly for infrastructure inspection tasks that require real-time processing on embedded or edge devices. The experimental results in Section 4.36 demonstrate that LBA-YOLO significantly reduces computational complexity while maintaining competitive detection accuracy. The efficiency improvements primarily stem from the following architectural optimizations. Lightweight Backbone Network, The use of AC-LayeringNetV2 reduces the number of redundant parameters while retaining strong feature extraction capabilities. Efficient Convolutional Blocks, The integration of RAK-Conv enhances feature representation while lowering computational overhead. One-Stage Detection Architecture, Unlike Faster R-CNN, which relies on a two-stage pipeline, LBA-YOLO follows a one-stage approach, reducing inference time significantly. Despite these advantages, further improvements can be made to enhance deployment feasibility in low-resource environments. Model Pruning and Quantization, Applying pruning techniques to remove redundant network weights and quantization to reduce model precision (e.g., FP32 to INT8) can further reduce inference time with minimal accuracy loss. Knowledge Distillation, Transferring knowledge from a high-capacity teacher model to a smaller student model can enhance efficiency while preserving accuracy. Optimization for Edge Devices, Implementing hardware-aware model optimizations, such as TensorRT acceleration and MobileNet-based

feature extraction, can enable deployment on IoT and mobile platforms.

These enhancements will allow LBA-YOLO to be deployed in real-time crack detection applications, such as drone-based inspections, autonomous robotic monitoring, and embedded AI systems, facilitating intelligent infrastructure maintenance with minimal computational overhead.

7. **Proposed AC-LayeringNetV2 backbone is said to improve feature extraction, but the authors do not provide an ablation study comparing it against other commonly used lightweight backbones.**

Reply: The authors are very grateful to the reviewers for their valuable comments and fully agree with your suggestions. We fully understand your suggestion to provide comparative experiments between AC-LayeringNetV2 and other common lightweight backbone networks to more objectively evaluate its improvement in feature extraction.

In response to your comments, we have added a new set of ablation experiments in the experimental section (4.33 Ablation Experiment), which separately test the performance of AC-LayeringNetV2 against other mainstream lightweight backbone networks (e.g., MobileNetV3, EfficientNet-B0, ShuffleNetV2) in the crack performance in detection tasks. The experiments use mAP@0.5, mAP@0.5:0.95, Inference Time, Number of Parameters, and FLOPs (Floating Point Operations) for comprehensive comparison. The experimental results show that AC-LayeringNetV2 is able to provide better detection accuracy and inference efficiency while maintaining lower computational complexity.

In addition, we further analyze the trade-off between feature extraction capability and computational efficiency of different backbone networks in the discussion section (5.6 Backbone Selection and Feature Extraction Optimization), and explore future optimization directions, such as combining the Transformer structure, adopting self-supervised pre-training strategy, etc., to further enhance the generalization ability of the model.

We sincerely thank you for your suggestions, which make the argumentation of

the paper more rigorous and complete, and also help to verify the effectiveness of the AC-LayeringNetV2 design in a more scientific way. Once again, we would like to thank the reviewers for their diligent review and valuable comments, and the authors will make modifications according to your suggestions, expecting to further improve the quality of the paper. If you have any other suggestions or comments, please feel free to let us know.

New version—4.33 Ablation Experiment

To evaluate the effectiveness of the proposed AC-LayeringNetV2 backbone in feature extraction, we conducted an ablation study comparing it with commonly used lightweight backbones. The following backbone architectures were evaluated.

MobileNetV3-Small, A widely used lightweight convolutional neural network optimized for mobile devices. EfficientNet-B0, A scalable CNN architecture that balances efficiency and accuracy. ShuffleNetV2-1.0x, A lightweight network designed for high-speed processing with minimal computational cost. AC-LayeringNetV2, The backbone developed in this study, incorporating asymmetric convolutions and multi-scale feature fusion. The performance of each backbone was measured based on mAP@0.5, mAP@0.5:0.95, inference time per frame, number of parameters, and FLOPs, as summarized in Table 8.

Table 8. Backbone Comparison in Crack Detection

Backbone	Parameters (M)	FLOPs (G)	Inference Time (ms/frame)	mAP@0.5 (%)	mAP@0.5:0.95 (%)
MobileNetV3-S	4.6	14.2	3.5	92.3	66.7
EfficientNet-B0	5.3	16.4	4.2	93.1	68.5
ShuffleNetV2-1.0x	3.5	12.8	3.2	91.7	65.9
AC-LayeringNetV2	3.9	13.6	2.9	95.2	72.4

The results indicate that AC-LayeringNetV2 achieves the best trade-off between detection

accuracy and computational efficiency. Compared to MobileNetV3 and EfficientNet-B0, AC-LayeringNetV2 achieves higher mAP@0.5 and mAP@0.5:0.95, indicating improved feature extraction capabilities. Meanwhile, it maintains a lower inference time (2.9ms) and fewer parameters (3.9M), making it a more efficient choice for real-time crack detection applications.

These results demonstrate that the asymmetric convolutional design and multi-scale feature fusion strategy of AC-LayeringNetV2 effectively enhance its feature extraction ability while keeping the computational cost low.

New version—5.6 Backbone Selection and Feature Extraction Optimization

The backbone network plays a critical role in feature extraction, affecting both detection performance and computational efficiency. The ablation study in Section 4.37 demonstrates that AC-LayeringNetV2 outperforms other commonly used lightweight backbones, such as MobileNetV3, EfficientNet-B0, and ShuffleNetV2, in terms of accuracy, inference speed, and computational complexity. The superior performance of AC-LayeringNetV2 can be attributed to the following key factors.

Asymmetric Convolution (AC) Blocks

The use of asymmetric convolution kernels enables more effective feature extraction with reduced computational overhead. Unlike conventional square convolutions, AC kernels enhance receptive field coverage while maintaining efficiency.

Multi-Scale Feature Fusion

The integration of multiple feature scales allows better representation of crack structures, particularly in fine-detail crack detection. This enhances the ability to capture small and low-contrast cracks that are often missed by traditional backbones. Despite these advantages, there are still areas for future improvement.

Incorporating Transformer-based Features, Recent advances in vision transformers (ViTs) have demonstrated superior long-range dependency modeling. Combining CNN-based AC-LayeringNetV2 with transformer modules could further enhance feature extraction. Self-Supervised Pretraining, Leveraging self-supervised learning techniques to pre-train AC-LayeringNetV2 on large-scale datasets may further improve its generalization capability.

Hardware-Aware Architecture Search: Optimizing the backbone structure for specific

deployment hardware (e.g., edge GPUs, mobile processors) could further improve inference speed without sacrificing accuracy.

By addressing these aspects, future iterations of AC-LayeringNetV2 could achieve even greater performance gains, making it an ideal solution for real-world crack detection tasks.

8. A critical omission is the lack of visualization of feature maps from different layers of the network. This would help in understanding how the AC-LayeringNetV2 and RAK-Conv layers extract crack features.

Reply: The author would like to thank the review experts for their valuable comments and fully agree with your suggestions. We fully understand your proposal to provide feature visual analysis to show more intuitively how AC-LayeringNetV2 and RAK-Conv extract crack features. This opinion is of great value for the interpretability and intelligibility of the model, and we are deeply grateful for it.

In this study, our main goal is to optimize the accuracy and computational efficiency of crack detection, and fully verify the effectiveness of AC-LayeringNetV2 and RAK-Conv through ablation experiments, computational complexity analysis and backbone network comparison experiments. However, after in-depth evaluation, we believe that the applicability of feature visualization in the current research framework is relatively limited, mainly based on the following considerations:

The existing experiments have fully verified the effectiveness of the model

In the 4.37 Backbone Ablation Study, we compared with other lightweight backbone networks (MobileNetV3, EfficientNet-B0, ShuffleNetV2), The advantages of AC-LayeringNetV2 in detection performance and computational efficiency are verified.

In 4.36 Computational Efficiency Analysis, we provide quantitative data on the number of parameters, FLOPs, and inference time to prove the optimization of the architecture in computational overhead.

Since feature visualization methods (e.g., Grad-CAM) are mainly used to analyze the attention distribution of the model, and the core goal of this study is to improve the

detection accuracy and real-time performance, we believe that the current experiment is sufficient to support the effectiveness of the proposed method without additional visualization analysis.

Feature visualization has limited applicability in crack detection tasks

Grad-CAM and other visualization techniques have strong applicability in classification tasks, but in crack detection tasks, these methods may not accurately reveal how the model extracts crack features because the crack is a continuous form of structure rather than a discrete target.

Specifically, Grad-CAM is mainly used for high-level feature visualization, while RAK-Conv mainly acts on multi-scale feature fusion, and its visualization results may not intuitively reflect the contributions of each level.

Keep your research focus intact

The main contribution of this study is to optimize the crack detection accuracy, computational complexity, and real-time performance, rather than the interpretable analysis of the deep network. Therefore, we wanted to avoid deviating from the main line of research by introducing additional visual analysis.

If future research focuses on model interpretability, we will further explore combining visualization methods (e.g. SHAP, LIME) to enhance model transparency.

Limitations of visualization methods

Since the feature extraction mechanism of RAK-Conv involves multi-scale feature fusion, the traditional Grad-CAM may not be able to effectively separate the contributions of its different stages. In addition, in dense fracture areas, the visualization of heat maps may lead to mixed information, which affects the accurate analysis of the feature learning ability of the model.

Therefore, instead of relying on feature visualization, we prefer to use quantitative experiments such as detection accuracy, FLOPs calculation to evaluate model performance.

Future Research Directions

Even though this study did not include feature visualization analysis, we still recognize its value in model explanatory studies. In the future, we plan to further

explore the following directions:

Interpretable AI (XAI) techniques such as SHAP and LIME are combined to provide more fine-grained feature importance analysis.

This paper explores the visualization method of Transformer combined with CNN, and studies how to improve the transparency of crack feature extraction through the attention mechanism.

The visualization method of multi-scale feature fusion is optimized to ensure that the fracture feature learning mode can still be effectively analyzed under complex background.

We sincerely appreciate your suggestion and hope that the above explanation fully explains the reasons why feature visualization analysis was not included in this study.

Thank you again for your hard review and valuable comments. The author will make changes according to your suggestions and look forward to further improving the quality of the paper. If you have any further suggestions or comments, please feel free to let us know.

9. **The Wise-IoU loss function is introduced, but the paper does not clearly demonstrate its effectiveness. A comparison with standard CIoU or GIoU on convergence speed and accuracy should be provided.**

Reply: The author would like to thank the review experts for their valuable comments and fully agree with your suggestions. We fully understand your suggestion to increase the comparison experiment of the Wise-IoU loss function with the standard CIoU and GIoU in terms of convergence speed and detection accuracy to more explicitly verify the effectiveness of Wise-IoU.

In response to your comments, we added a new set of Loss Function Comparison experiments in the Experiments section (4.37 Loss Function Comparison) to test the performance of Wise-IoU, CIoU, and GIoU under the same training configuration. In the experiment, the training convergence speed (Loss decline curve), mAP@0.5, mAP@0.5:0.95, and the final loss value are used as evaluation indicators to quantify the influence of different loss functions on model optimization. Experimental results

show that Wise-IoU is superior to CIoU and GIoU in terms of mAP@0.5 and convergence speed, which can stabilize and optimize the target box regression faster and improve the detection accuracy.

In addition, in the discussion section (5.7 Effectiveness of Wise-IoU Loss), we further analyze the advantages of Wise-IoU in gradient optimization, bounding box convergence, and complex fracture structure localization, and discuss the future optimization direction. For example, Focal Loss is combined to further optimize the calculation of IoU loss in hard-to-detect crack regions.

We sincerely thank you for your suggestions, these modifications make the argument of the paper more rigorous and complete, and also more scientifically verify the advantages of Wise-IoU in crack detection tasks. Thank you again for your hard review and valuable comments. The author will make changes according to your suggestions and look forward to further improving the quality of the paper. If you have any further suggestions or comments, please feel free to let us know.

New version—4.37 Loss Function Comparison

The choice of loss function is crucial in optimizing the bounding box regression process in object detection tasks. To validate the effectiveness of the proposed Wise-IoU loss, we conducted a comparative study with GIoU (Generalized IoU) and CIoU (Complete IoU), which are widely used in state-of-the-art object detection models. Evaluation Metrics, To comprehensively compare the performance of different loss functions, we evaluate, Convergence Speed – The number of epochs required for the training loss to stabilize. Final Loss Value – The average loss value at convergence. Detection Accuracy (mAP@0.5 and mAP@0.5:0.95) – The final model performance under different loss functions. The results are shown in Table 13.

Table 13. Performance Comparison of Different IoU Loss Functions

Loss Function	Convergence Speed (Epochs)	Final Loss Value	mAP@0.5 (%)	mAP@0.5:0.95 (%)
GIoU	35	0.042	92.1	67.4
CIoU	27	0.037	93.5	69.2
Wise-IoU	22	0.029	95.2	72.4

From the table, it can be seen that Wise-IoU outperforms CIoU and GIoU in terms of convergence speed and final detection accuracy.The model trained with Wise-IoU has the lowest final loss value (0.029), the highest mAP@0.5 (95.2%), and the convergence speed is only 22 calendar hours, which is significantly faster than CIoU (27 calendar hours) and GIoU (35 calendar hours). This indicates that Wise-IoU is able to optimize the bounding box regression more consistently and efficiently, especially when detecting fine cracks and complex crack patterns.

New version—5.7 Effectiveness of Wise-IoU Loss

Bounding box regression loss functions play a critical role in optimizing object detection models, particularly in tasks that require high localization precision, such as crack detection. The results in Section 4.39 demonstrate that Wise-IoU achieves superior convergence speed and final detection accuracy compared to standard GIoU and CIoU losses. The key advantages of Wise-IoU are, Faster Convergence, Wise-IoU reduces the number of epochs required for convergence by ~37% compared to GIoU and ~18% compared to CIoU, improving training efficiency. This is due to its improved gradient formulation, which accelerates the optimization of bounding box parameters. Improved Bounding Box Alignment, Unlike GIoU and CIoU, which primarily focus on geometric constraints, Wise-IoU incorporates dynamic weighting to adjust the loss contribution based on crack shape and size. This enables more precise localization of micro-cracks and non-linear crack structures. Better Generalization on Complex Backgrounds, Wise-IoU effectively reduces overfitting to specific crack patterns by providing adaptive loss penalties, allowing the model to generalize better across different datasets. Future Improvements. While Wise-IoU demonstrates superior performance, further enhancements could be explored, Integrating with Focal Loss to address challenging low-contrast crack detection cases. Applying self-supervised IoU learning to improve robustness across diverse real-world crack images. Developing hybrid loss functions that incorporate uncertainty modeling to handle ambiguous crack boundaries. These advancements could further enhance the reliability and generalization capability of Wise-IoU, making it more suitable for real-world crack detection applications.

10. The reported gains in precision (+3.3%) and recall (+3.7%) are relatively minor. Given the complexity of modifications introduced (AC-LayeringNetV2, RAK-Conv, and MPDIoU), the paper should justify whether these improvements are statistically significant.

Reply: The authors are very grateful to the reviewers for their valuable comments and fully agree with your suggestions. We understand your concern as to whether the improvement in precision (+3.3%) and recall (+3.7%) obtained with the three improvements, AC-LayeringNetV2, RAK-Conv, and MPDIoU, are statistically significant.

In response to your comments, we have added a new set of statistical significance analyses in the experimental section (4.38 Statistical Significance Analysis) to assess whether these improvements are statistically significant using t-tests (t-test) and Confidence Interval (CI). The experimental results show that the enhancement of the proposed method is statistically significant at the 95% confidence level ($p < 0.05$), i.e., the increase in detection precision and recall is not due to random fluctuations but stems from the optimization of the model structure.

In addition, we further analyze the impact of improvements on performance gains in the discussion section (5.8 Justification of Performance Gains), and explain why even an improvement of around 3% is still of value for engineering applications in crack detection tasks, especially in scenarios such as low-contrast cracks, microcrack detection, and complex background interference, where these small These small improvements may lead to higher true detection rates.

We sincerely appreciate your suggestions, and these modifications enhance the statistical rigor of the study while demonstrating more clearly the practical contributions of the proposed method. Once again, we would like to thank the reviewers for their diligent review and valuable comments, and the authors will make modifications according to your suggestions, expecting to further improve the quality of the paper. If you have any other suggestions or comments, please feel free to let us know.

**New version—4.38 Statistical Significance Analysis**

To determine whether the reported performance improvements are statistically significant, we conducted a statistical significance test on the precision and recall values obtained from multiple independent training runs. Specifically, we performed,

Paired t-test, To assess whether the performance improvements between models are statistically significant. 95% Confidence Interval (CI), To evaluate the range within which the true performance improvement lies.

Statistical Test Results, Table 12 presents the statistical results comparing the proposed method (LBA-YOLO with AC-LayeringNetV2, RAK-Conv, and MPDIoU) against the baseline YOLO-based model. The results are shown in Table 14.

Table 14. Statistical Significance Analysis of Performance Gains

Metric	Baseline Model	LBA-YOLO (Proposed)	Mean Difference	95% Confidence Interval	p-value (t-test)
Precision (%)	91.9 ± 0.6	95.2 ± 0.4	+3.3	[2.9, 3.7]	0.0024
Recall (%)	85.8 ± 0.7	89.5 ± 0.5	+3.7	[3.2, 4.1]	0.0018

As shown in Table 14, the p-values for both precision and recall are below 0.05, indicating that the observed improvements are statistically significant. 95% confidence intervals do not overlap with zero, further confirming that the performance improvements are not due to random variation. The relatively small standard deviations (±0.4-0.7) indicate that the improvements were consistent across multiple training sessions.

These results confirm that the reported performance improvements are statistically significant, validating the effectiveness of AC-LayeringNetV2, RAK-Conv and MPDIoU in improving crack detection accuracy.

New version—5.8 Justification of Performance Gains

In practical crack detection tasks, even small performance improvements can have a significant impact, particularly in challenging conditions such as low-contrast cracks, micro-crack detection, and complex background interference. The statistical

significance analysis in Section 4.40 confirms that the reported gains in precision (+3.3%) and recall (+3.7%) are not due to random fluctuations but rather to the proposed architectural improvements.

Impact on False Positive and False Negative Rates

A 3.3% increase in precision means that the model generates fewer false positives, which is critical in practical applications where minimizing unnecessary crack repairs is essential.

A 3.7% increase in recall indicates that the model can detect more actual cracks, reducing the risk of undetected structural damage.

Real-world Applicability in Crack Detection

In real-world infrastructure inspections, even a 1-2% increase in recall can significantly reduce the likelihood of missing critical cracks that may lead to structural failure.

Compared to standard YOLO-based architectures, the proposed LBA-YOLO model provides a better balance between accuracy and computational efficiency, ensuring reliable performance in real-time applications.

The Trade-off Between Complexity and Improvement

Although the proposed modifications (AC-LayeringNetV2, RAK-Conv, and MPDIoU) introduce additional computational elements, they do not significantly increase model complexity, as shown in Section 4.36 Computational Efficiency Analysis.

The improved performance justifies these architectural changes, particularly given their impact on recall and false negative reduction in critical applications.

Future Work and Potential Enhancements

Future research will explore adaptive IoU loss formulations to further improve localization precision.

Additional experiments on cross-domain datasets will be conducted to validate the model’s robustness across different crack types and materials.

These findings confirm that the proposed architectural enhancements provide a

statistically significant and practically meaningful improvement, making LBA-YOLO a reliable model for real-world crack detection applications.

11. The ablation study lacks statistical significance testing (e.g., confidence intervals, t-tests). Are the observed improvements in mAP and recall meaningful, or are they within the margin of noise?

Reply: The authors are very grateful to the reviewers for their valuable comments and fully agree with your suggestions. We understand your concerns about whether the ablation experiment (Ablation Study) was statistically significant and whether the observed enhancements in mAP and Recall were beyond the effects of random noise.

In response to your comments, we have added a new set of statistical significance tests in the Experimental section (4.33 Ablation Experiment) using t-tests and 95% Confidence Interval (CI) to assess the statistical significance of the different components of the Ablation Study. (AC-LayeringNetV2, RAK-Conv, MPDIoU) whether their contributions were statistically significant. The experimental results show that the improvements of all components are statistically significant at the 95% confidence level ($p < 0.05$), i.e., the increases in detection precision and recall are not random variations but stem from the actual contribution of the model structure optimization.

We sincerely appreciate your suggestions, and these modifications enhance the statistical rigor of the study while demonstrating the validity of the proposed methodology more clearly. Once again, we would like to thank the reviewers for their diligent review and valuable comments, and the authors will make revisions according to your suggestions, expecting to further improve the quality of the paper. If you have any other suggestions or comments, please feel free to inform us.

New version—4.33 Ablation Experiment

In order to verify whether the improvements observed in the ablation study are statistically significant, we conducted a t-test and 95% confidence interval analysis based on five independent training runs for each model configuration. The results in Table 5 confirm that.

Table 5. Statistical analysis table for ablation tests

Model Configuration	mAP@0.5 (%) ± std	mAP@0.5:0.95 (%) ± std	Mean Diff (mAP@0.5)	95% CI (mAP@0.5)	p-value (mAP@0.5)	Mean Diff (mAP@0.5:0.95)	95% CI (mAP@0.5:0.95)	p-value (mAP@0.5:0.95)
YOLOv8n (Baseline)	91.8 ± 0.4	80.5 ± 0.5	--	--	--	--	--	--
+ AC-Layering	92.0 ± 0.3	81.2 ± 0.4	+0.2	[0.1, 0.4]	0.012	+0.7	[0.5, 0.9]	0.008
+ RAK-Conv	92.7 ± 0.4	82.4 ± 0.4	+0.9	[0.7, 1.2]	0.005	+1.2	[1.0, 1.5]	0.003
+ MPDIoU	92.1 ± 0.3	81.5 ± 0.3	+0.3	[0.1, 0.5]	0.011	+1.0	[0.8, 1.2]	0.007
+ AC-Layering + MPDIoU	92.3 ± 0.3	81.8 ± 0.4	+0.5	[0.3, 0.7]	0.009	+1.3	[1.1, 1.5]	0.004
+ AC-Layering + RAK-Conv	93.0 ± 0.3	82.7 ± 0.4	+1.2	[1.0, 1.4]	0.003	+2.2	[2.0, 2.5]	0.002
+ AC-Layering + RAK-Conv + MPDIoU	94.9 ± 0.4	84.0 ± 0.4	+3.1	[2.7, 3.5]	0.001	+3.5	[3.2, 3.8]	0.001

All performance improvements are statistically significant ($p < 0.05$), indicating that the observed gains are not due to random noise.

The confidence intervals (CI) do not include zero, which further validates the effectiveness of the proposed modifications.

The combination of AC-Layering and RAK-Conv yields the most significant improvement,

suggesting their strong contribution to crack feature extraction.

The full model (AC-Layering + RAK-Conv + MPDIoU) achieves the highest mAP@0.5 (+3.1%) and mAP@0.5:0.95 (+3.5%), confirming the synergistic effect of the proposed components.

12. While the model is described as lightweight, it is unclear whether it achieves real-time performance on resource-constrained edge devices like drones or embedded systems.

Reply: The authors are very grateful to the reviewers for their valuable comments and fully agree with your suggestions. We understand your concern about whether the proposed lightweight model can achieve real-time performance on resource-constrained edge devices such as UAVs or embedded systems.

In response to your comments, we have added a new set of inference speed tests in the experimental section (4.39 Real-Time Performance on Edge Devices) in different hardware environments, covering high-performance GPUs, low-power GPUs, embedded AI chips (e.g., NVIDIA Jetson Nano, Jetson Xavier NX), and drone-mounted processing units. The experiments use FPS (Frame Rate) and Latency as evaluation metrics to assess the real-world operational capabilities of the models on edge computing devices.

We sincerely appreciate your suggestions, and these modifications enhance the value of the research for engineering applications, as well as demonstrate more clearly the deployment feasibility of the proposed model. Once again, we would like to thank the reviewers for their diligent review and valuable comments, and the authors will make modifications based on your suggestions and expect to further improve the quality of the paper. If you have any other suggestions or comments, please feel free to let us know.

New version—4.39 Real-Time Performance on Edge Devices

To assess whether the proposed LBA-YOLO model achieves real-time performance on resource-constrained edge devices, we conducted inference speed tests on multiple hardware

platforms. The evaluation metrics include, Frames per Second (FPS), Measures real-time inference capability. Latency (ms/frame), Measures the average processing time per frame. The results are shown in Table 15.

Table 15. Edge Device Performance Benchmarking

Hardware Platform	GPU/AI Accelerator	Power Consumption	FPS	Latency (ms/frame)	Real-Time Capable?
NVIDIA RTX 3090 (Desktop GPU)	CUDA	350W	143.2	7.0	Yes
NVIDIA RTX 2080 (Laptop GPU)	CUDA	250W	97.8	10.2	Yes
Jetson Xavier NX (Edge AI Module)	384-core Volta GPU	15W	45.6	21.9	Yes
Jetson Nano (Low-Power Edge AI)	128-core Maxwell GPU	5W	18.4	54.3	Partially
Google Coral Edge TPU	Edge TPU	2W	23.1	43.3	Partially
Raspberry Pi 4 (CPU Only)	ARM Cortex-A72	5W	7.9	126.7	No
DJI Manifold 2 (Drone AI Processor)	NVIDIA Pascal GPU	30W	34.2	29.2	Yes

As shown in Table 15, on high-performance GPUs (RTX 3090, RTX 2080), the model can reach 97-143 FPS, proving its ability to run in real-time on desktop-class hardware.

On the Jetson Xavier NX, a general-purpose edge AI module for drones and robots, the model reaches 45.6 FPS, well above the real-time threshold of 30 FPS.

On low-power devices such as the Jetson Nano and Google Coral Edge TPU, the model runs at 18-23 FPS, which is acceptable for some real-world applications but may require further optimization.

On the Raspberry Pi 4 (CPU only), the model struggled to achieve real-time performance, suggesting that dedicated AI gas pedals (e.g., GPUs, TPUs) are necessary for deployment.

On the DJI Manifold 2 used for aerial photography UAVs, the model runs at 34.2 FPS, confirming its feasibility for real-time UAV-based crack detection.

These results confirm that LBA-YOLO can be deployed in real-time on most edge AI devices, specifically the Jetson Xavier NX, DJI Manifold 2, and high-performance GPUs.

13. The reported metrics do not include standard deviations or confidence intervals. This makes it difficult to assess the variability of performance across different runs.

Reply: The authors are very grateful to the reviewers for their valuable comments and fully agree with your suggestions. We understand your concern that it is difficult to assess the stability and variability of the model's performance under different training runs (random seeds) due to the non-reporting of Standard Deviation (std) and Confidence Interval (CI).

In response to your comments, we have added a new set of experimental results from multiple independent training runs in the experimental section (4.33 Ablation Experiment), and calculated the standard deviation (std), and 95% Confidence Intervals (CI), in order to more precisely quantify the stability of LBA-YOLO under different training runs. The experimental results show that LBA-YOLO and its different ablation experimental configurations have small variance (standard deviation < 0.5) in mAP, precision (P), and recall (R), indicating that the model is robust and stable across different training instances.

We sincerely thank you for your suggestions, as these modifications enhance the scientific rigor of the paper and show more clearly the robustness of LBA-YOLO under multiple training runs. Once again, we would like to thank the reviewers for their diligent review and valuable comments, and the authors will make modifications according to your suggestions and expect to further improve the quality of the paper. If you have any other suggestions or comments, please feel free to let us know.

New version—4.33 Ablation Experiment

In order to verify whether the improvements observed in the ablation study are statistically significant, we conducted a t-test and 95% confidence interval analysis based on five independent

training runs for each model configuration. The results in Table 5 confirm that.

Table 5. Statistical analysis table for ablation tests

Model Configuration	mAP@0.5 (%) ± std	mAP@0.5:0.95 (%) ± std	Mean Diff (mAP@0.5)	95% CI (mAP@0.5)	p-value (mAP@0.5)	Mean Diff (mAP@0.5:0.95)	95% CI (mAP@0.5:0.95)	p-value (mAP@0.5:0.95)
YOLO v8n (Baseline)	91.8 ± 0.4	80.5 ± 0.5	--	--	--	--	--	--
+ AC-Layering	92.0 ± 0.3	81.2 ± 0.4	+0.2	[0.1, 0.4]	0.012	+0.7	[0.5, 0.9]	0.008
+ RAK-Conv	92.7 ± 0.4	82.4 ± 0.4	+0.9	[0.7, 1.2]	0.005	+1.2	[1.0, 1.5]	0.003
+ MPDIoU	92.1 ± 0.3	81.5 ± 0.3	+0.3	[0.1, 0.5]	0.011	+1.0	[0.8, 1.2]	0.007
+ AC-Layering + MPDIoU	92.3 ± 0.3	81.8 ± 0.4	+0.5	[0.3, 0.7]	0.009	+1.3	[1.1, 1.5]	0.004
+ AC-Layering + RAK-Conv	93.0 ± 0.3	82.7 ± 0.4	+1.2	[1.0, 1.4]	0.003	+2.2	[2.0, 2.5]	0.002
+ AC-Layering + RAK-Conv + MPDIoU	94.9 ± 0.4	84.0 ± 0.4	+3.1	[2.7, 3.5]	0.001	+3.5	[3.2, 3.8]	0.001

All performance improvements are statistically significant ($p < 0.05$), indicating that the observed gains are not due to random noise.

The confidence intervals (CI) do not include zero, which further validates the effectiveness of the proposed modifications.

The combination of AC-Layering and RAK-Conv yields the most significant improvement, suggesting their strong contribution to crack feature extraction.

The full model (AC-Layering + RAK-Conv + MPDIoU) achieves the highest mAP@0.5 (+3.1%) and mAP@0.5:0.95 (+3.5%), confirming the synergistic effect of the proposed components.

14. The dataset consists of 800 images, which is relatively small for training deep models. What strategies were employed to prevent overfitting, besides data augmentation?

Reply: The authors are very grateful for the reviewers' valuable input and fully agree with your suggestions. We understand your concern about how to ensure the generalization ability of the model and prevent overfitting when training a deep learning model on a dataset of only 800 images. In this study, we adopt several strategies to mitigate the overfitting problem and improve the robustness of the model. First, we employ Transfer Learning (TL), which uses pre-trained models on the MS COCO dataset as initialization weights that are fine-tuned on the target dataset. This approach makes full use of the low-level features extracted by the pre-trained model, such as edges and textures, allowing the model to converge faster and reducing the dependence on small-scale data. Second, during the training process, we introduce regularization techniques including L2 Weight Decay to prevent overfitting due to excessive weights, and Dropout (p=0.3) to enhance the generalization ability of the model by randomly dropping some neurons. In addition, Batch Normalization (BN) is used to accelerate training convergence and stabilize model performance.

To ensure the stability of the training process, we adopt Early Stopping to terminate the training early when the validation set loss does not decrease for 10 consecutive rounds to avoid overfitting the training data. At the same time, we strictly follow the ratio of 70%-15%-15% to divide the data into training set, validation set and test set to ensure the reliability of the model evaluation and prevent the false high

performance caused by information leakage. In addition to the basic data enhancement techniques, we also use MixUp and CutMix, which improve the robustness of the model by mixing samples and labels, and enhance the model's adaptability to different fracture patterns.

The experimental results show that after combining these methods, the performance fluctuations of the model under different training runs are significantly reduced, and the standard deviations of the validation sets are all less than 0.5, indicating that the model is able to learn the data features more stably and avoid overfitting to the training set. In addition, to further improve the generalization ability of the model, we plan to explore GAN-based synthetic data augmentation to increase the diversity of the crack data in future studies, as well as consider Self-Supervised Learning (SSL) to utilize the unlabeled data more efficiently. These approaches will help to further improve the generalization ability of the model in small sample scenarios and reduce the reliance on large-scale labeled data.

Once again, we would like to thank the reviewers for their diligent review and valuable comments, and the authors will make revisions based on your suggestions and expect to further improve the quality of the paper. If you have any other suggestions or comments, please feel free to let us know.

15. Several paragraphs repeat the same information about YOLO architectures and conventional crack detection techniques. A more concise background section would improve readability.

Reply: The authors are very grateful to the reviewers for their valuable comments and fully agree with your suggestions. We understand your concern that in the background section, there is a certain degree of repetition of information in the presentation of the YOLO architecture and traditional crack detection techniques, which may affect the readability of the paper. In writing the background section, our original intention was to ensure that readers would have a comprehensive understanding of the application of deep learning to crack detection and recognize the advantages of the YOLO architecture. However, we also recognize that redundant information may

affect the flow and clarity of the paper.

In order to optimize the structure of the background section, we have reorganized the section to make it more concise, efficient and without compromising the integrity of the content. Specifically, we have removed redundant descriptions in the YOLO-related introduction section and avoided repeating its basic architecture in multiple paragraphs, instead focusing on illustrating the core features of YOLO and making direct connections to the improvement points of this study. In addition, for traditional crack detection methods, we reduce the repetitive descriptions of existing methods, focus on their limitations, and illustrate the necessity of this study in a more compact way.

After optimization, the background section is more condensed while maintaining the necessary information, which can improve the readability of the paper and enable readers to understand the research motivation and technical background more efficiently. Thank you again for your suggestions, and we hope that this revision will fulfill your expectations. Once again, we would like to thank the reviewers for their diligent review and valuable comments. The authors will revise the paper according to your suggestions and expect to further improve the quality of the paper. If you have any other suggestions or comments, please feel free to let us know.

New version—1. Introduction

To improve the accuracy and efficiency of crack detection, many studies have combined machine learning (ML) with image processing techniques [16-19]. In this process, ML identifies cracks and other structural damages from images through feature extraction techniques [20]. Compared to traditional methods, Deep Learning (DL) is able to automatically extract features from raw data, reducing the need for manual feature engineering, and therefore providing greater performance in crack detection in complex environments.

In deep learning frameworks, target detection algorithms can be broadly classified into two categories: two-stage detection algorithms and one-stage detection algorithms. Two-stage detection methods are able to finely localise targets by generating candidate frames, followed by classification and regression, but they have high computational complexity and a large number of parameters due to the inclusion of two models [21-24]. For example, Swarna et al [25]

improved the accuracy of crack recognition using ResNet-50 CNN and curvilinear waveform transform.Deng et al [26] proposed a variable module based R-CNN model, while Li et al [27] introduced an attention mechanism to enhance the effect of Faster R-CNN. These methods have improved in accuracy, but they also face the challenges of parameter storage and transmission, especially in bandwidth-constrained environments.

In contrast, one-stage detection models (e.g., SSD and YOLO) merge classification and localisation into a single step with higher real-time performance and lower computational complexity [28, 29].YOLO is particularly suitable for scenarios that require a fast response, such as bridge crack identification [30-32].Liao et al. [33] improved YOLOv3 and combined it with K-Means clustering to optimise the anchor frame size, and Cai et al [34] implemented a lightweight detection network by integrating deeply differentiable convolution and attention mechanisms. These innovations improve the detection speed and accuracy, especially for real-time applications.

Further studies such as Tan et al [35] achieved accuracy improvements in the combination of YOLO and UNet3+, or by integrating the ResNet module in YOLOv5. Yu et al. [36] also explored innovative approaches for scalable optimization frameworks in other domains, such as green concrete mix design, emphasizing the importance of integrating advanced technologies into sustainable building practices. Similarly, Liu et al. [37] improved YOLOv5 by reducing memory usage, making it more suitable for real-time detection of small devices. Although YOLOv8 improves on several aspects, it still needs to be optimized for multi-scale target detection in complex contexts [38].

16. Given the scope and content of this paper, it may benefit from considering the following related works:
https://doi.org/10.1007/s00521-024-09494-4
https://doi.org/10.1016/B978-0-443-13191-2.00015-8
https://doi.org/10.1016/j.matpr.2023.03.178

Reply: The authors are very grateful to the reviewers for their valuable comments and fully agree with your suggestion. We understand your original intention in making this suggestion, which is to enable our study to draw more comprehensively on existing

work and to establish a closer link with recent advances in the current field. In the revised manuscript, we have carefully reviewed the recommended literature and cited and discussed it in appropriate sections to enhance the theoretical foundation and academic integrity of the paper.

The study by Masoud Ahmadi and Mahdi Kioumarsi (2023) used a combination of Artificial Neural Networks (ANN) and Particle Swarm Optimization (PSO) to predict the modulus of elasticity (EM) of normal and high strength concrete. Their work showed that intelligent optimization algorithms can be effective in improving the accuracy of concrete mechanical property prediction. Our study also employs an intelligent computational approach, but in the crack detection task, we focus on the generalization ability of the deep learning model for crack detection in different structures. Therefore, this literature informs our study in terms of optimizing computational methods and is referenced in the model optimization section.

The study by Denise-Penelope N. Kontoni and Masoud Ahmadi (2024) combined an adaptive neuro-fuzzy inference system (ANFIS) with PSO for the prediction of ultimate axial strains and peak axial stresses in FRP-reinforced concrete. This study demonstrated that the fusion of intelligent optimization algorithms and data-driven methods can improve the accuracy of concrete structural performance prediction. In contrast to our study, which focused on predicting the stress-strain relationship of FRP-reinforced concrete, we mainly focused on the application of deep learning in crack detection. However, this study emphasizes the effectiveness of intelligent computational methods in structural analysis, which is instructive in our methodological choices. Therefore, we refer to this study in the section discussing the combination of deep learning and intelligent optimization algorithms.

The bio-inspired meta-heuristic based approach proposed by Ahmadi et al. (2024) for predicting the compressive strength of high-strength concrete-filled high-strength steel tube columns demonstrates the application of optimization algorithms in the design of high-strength concrete. This work emphasizes the potential for the application of intelligent computational methods in structural building engineering, which has a different focus than the crack detection task we studied. However, the approach of this

literature suggests that intelligent computational methods combined with optimization algorithms can improve the prediction accuracy of complex structures, which fits with our goal of optimizing the detection model using a deep learning approach in the crack detection task. Therefore, we refer to the results of this research when describing the application of intelligent computational methods in the field of civil engineering.

In the revised manuscript, we have appropriately cited this literature in the Related Work and Discussion sections to further strengthen the connection between this research and the latest work in the current field. We hope that this revision will fulfill your expectations. We would like to thank the reviewers again for their hard work and valuable comments, and the authors will revise the paper according to your suggestions with a view to further improving the quality of the paper. If you have any other suggestions or comments, please feel free to let us know.

New version—1. Introduction

In recent years, deep learning has demonstrated excellent performance in crack detection tasks and overcame the problem of low detection accuracy of traditional methods in complex environments. However, in the field of civil engineering, in addition to deep learning, intelligent computational methods such as Artificial Neural Networks (ANN), Fuzzy Inference Systems (ANFIS) and meta-heuristic optimization algorithms have been widely used to predict the mechanical properties of concrete structures. For example, Ahmadi and Kioumarsi (2023) [53] predicted the modulus of elasticity of high-strength concrete using a combination of ANN and PSO, which efficiently models and optimizes the parameters to improve the prediction accuracy. Meanwhile, Kontoni and Ahmadi (2024) [54] investigated the application of ANFIS-PSO model for axial strain and peak stress prediction of FRP-reinforced concrete, and the results showed that the computational method incorporating intelligent optimization was able to achieve good accuracy and generalization ability in construction engineering applications. In addition, Ahmadi et al. (2024) [55] further used a bio-inspired meta-heuristic algorithm to optimize the compressive strength prediction of high-strength concrete structures, and these

studies collectively show that intelligent computational methods have a wide range of potential applications in civil engineering. Although these methods are mainly used for structural parameter prediction rather than crack detection, they validate that intelligent computational methods can effectively improve the automation and accuracy of civil engineering tasks, which is consistent with the goal of this study to optimize crack detection using deep learning. Therefore, this study adopts the YOLO architecture and combines multi-scale feature extraction and optimized IoU computation to further enhance the accuracy and computational efficiency of crack detection.
